# Paraneoplastic Internal Jugular Vein Thrombosis Leading to Diagnosis of Bilateral Ovarian Ependymoma

**DOI:** 10.1155/2014/324509

**Published:** 2014-05-21

**Authors:** Irappa Madabhavi, Apurva Patel, Mukesh Choudhary, Asha Anand

**Affiliations:** Department of Medical and Pediatric Oncology, GCRI, Civil Hospital Campus, Ahmedabad, Gujarat 830016, India

## Abstract

Ovarian ependymomas are extremely rare tumors of the ovary. We present a case of a 67-year-old lady presented to us with swelling in the right side of neck for 2 months followed by pelvic pain, lower abdominal distention, and weight loss for 1 month. Her coagulation profile, blood chemistry, lipid profile, and tumor markers were within normal limits. Neck Doppler ultrasonography revealed thrombus in the right internal jugular vein and CT scan of the abdomen showed bilateral ovarian masses. Patient was subjected to debulking surgery for suspected ovarian cancer and microscopy revealed a highly cellular tumor composed of small cells with hyperchromatic, round-to-oval nuclei with scanty cytoplasm, and perivascular pseudorosettes. Diagnosis was confirmed by immunophenotype showing strong positivity to glial fibrillary acidic protein, estrogen receptors, and progesterone receptors. Patient was successfully managed with anticoagulants, adjuvant chemotherapy with BEP regimen, and letrozole. After ruling out other common conditions for thrombosis in this age group, this seems to be a paraneoplastic presentation of ovarian malignancy that preceded the diagnosis of ependymoma by 2 months. To the best of our knowledge this is the first case report in the world literature as “paraneoplastic internal jugular vein thrombosis leading to diagnosis of bilateral ovarian ependymoma.”

## 1. Introduction

Thrombosis of the upper limbs and neck is very rare as compared to lower extremities. Internal jugular vein thrombosis is a very serious event, which can lead to pulmonary embolism and intracranial extension leading to intracranial thrombosis and cerebral edema. Patients usually present with painful swelling in the neck but sometimes may also be asymptomatic [1].

The increased risk for venous thrombosis in cancer has been considered an epiphenomenon. Paraneoplastic syndromes are attributed to tumor secretion of functional peptides and hormones or immune cross-reactivity between tumor and normal host tissues. Venous thromboembolism (VTE) and particularly idiopathic VTE may be paraneoplastic phenomena. Thromboembolic events are a major cause of morbidity in cancer patients and may be harbingers of occult malignancy. Appropriate recognition of the syndrome is paramount because VTE often requires careful medical surveillance and management. Ovarian malignancy may be silent even when it presents with venous gangrene [2].

Ependymoma is a glioma with differentiation toward ependymal cells that usually arises in the central nervous system. The histologic picture will be similar to that of ependymomas of the central nervous system. The diagnosis of ovarian ependymoma is usually supported by positive staining of cytoplasmic processes for glial fibrillary acidic protein. Ovarian ependymoma is extremely rare, and the treatment strategies for this disease have not been established. They have a favorable prognosis; patients with advanced stage disease are reported alive and well after treatment with surgery and chemotherapy [3].

However, recent studies from several laboratories have linked more closely malignant transformation (oncogenesis), tumor angiogenesis, and metastasis to the generation of clotting intermediates (e.g., tissue factor (TF), factor Xa, and thrombin), clotting or platelet function inhibitors (e.g., COX-2), or fibrinolysis inhibitors (e.g., plasminogen activator inhibitor, type 1 (PAI-10)) [4].

## 2. Case Presentation

A 67-year-old lady presented to us initially with an asymptomatic swelling in right side of neck for 2 months, which was insidious in onset and gradually progressive, followed by subacute pelvic pain, abdominal distension, and weight loss for 1 month. Patient had no history of surgery, central venous catheter insertion, any chemotherapeutic drug intake, diabetes, hypertension, or any ischemic disease in the past.

On examination her blood pressure, pulse rate, and respiratory rate were within normal ranges. Her height, weight, and body mass index were within normal range for her age. A small mass of size 2 × 2 cm was palpable in the right side of her neck which was superficial, movable, nontender, and nonpulsatile. On abdominal examination 5 × 6 cm and 7 × 7 cm masses were palpable on both sides of the lower abdomen with ascites. Bilateral adnexal masses were felt on gynecological examination.

Her blood chemistry, lipid profile, ANA profile, AFP, beta-HCG, and LDH were within normal limits. Coagulation profile including serum homocysteine levels, prothrombin time, activated partial thromboplastin time, fibrinogen levels, fibrinogen degradation products, D-dimer, protein-C, protein-S, and antithrombin III levels were within normal limits. The DNA of the patient from the peripheral blood was sent for genetic analysis, which did not show any mutations for Factor V Leiden G1691A (activated protein C resistance), prothrombin G20210A, or methylene tetrahydrofolate reductase (MTHFR) C677T. Anti-nuclear antibody (ANA), anti-cardiolipin antibody (ACA), and anti-beta2-glycoprotein I (GPI) by enzyme-linked immunosorbent assay (ELISA) and lupus anticoagulant (LA) assay tests were negative. Patient was subjected to venous Doppler of the neck and upper limb veins to rule out any local pathology.

**Figure 1:**
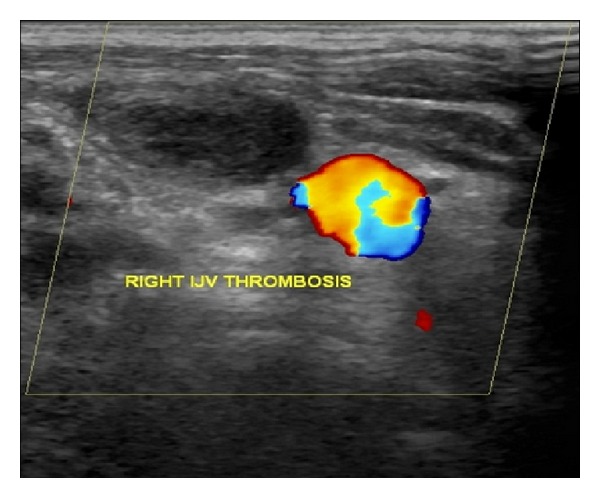
Doppler ultrasonography shows a thrombus in the right internal jugular vein.

**Figure 2:**
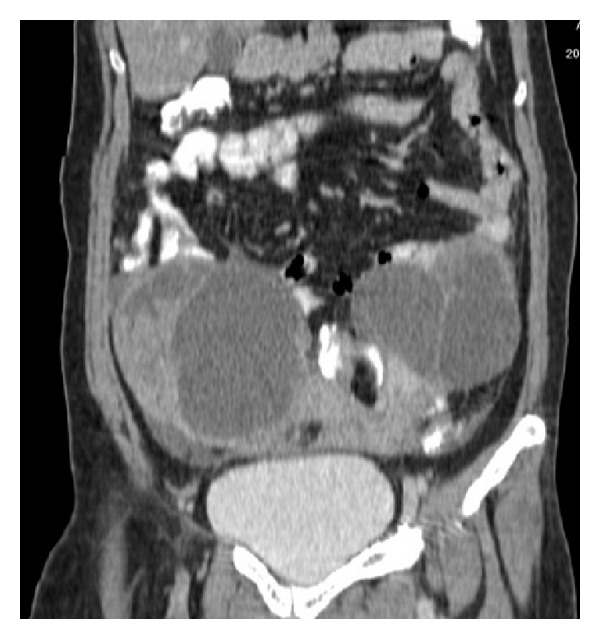
CT scan of the abdomen showing predominantly cystic lesions with internal septations and solid component which appeared after contrast enhancement in bilateral adnexal regions of size 98 × 88 × 107 mm on right side and 110 × 52 × 94 mm on left side.

Doppler ultrasonography revealed an expansile thrombus in the right internal jugular vein without any atherosclerotic plaque or calcification (Figure 1). Computed tomography (CT) image of the thorax also revealed right sided IJV thrombosis and there was no evidence of lung metastasis or mediastinal lymphadenopathy or any compressing mass lesions over IJV. CT scan of the abdomen showed predominantly cystic lesions with internal septations and solid component which appeared after contrast enhancement in bilateral adnexal regions of size 98 × 88 × 107 mm on right side and 110 × 52 × 94 mm on left side (Figure 2).

**Figure 3:**
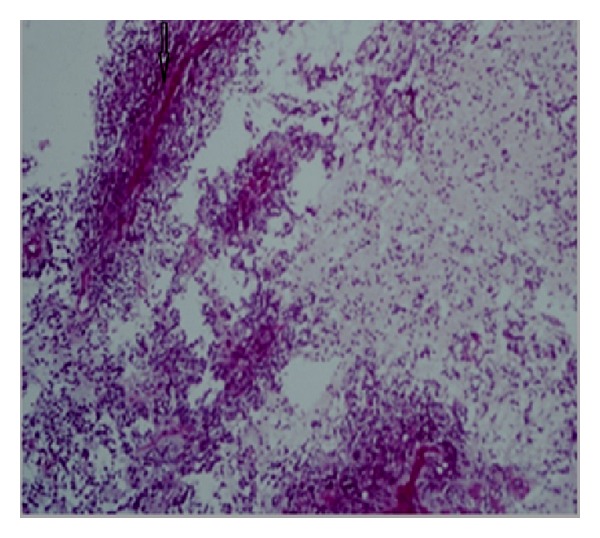
Histological section of ovary. Highly cellular tumor composed of small cells with hyperchromatic, round-to-oval nuclei, and scanty cytoplasm. Perivascular pseudorosettes (as depicted in the figure by an arrow mark), ependymal rosettes, and extensive necrosis.

Later the patient was subjected to optimal debulking surgery including total hysterectomy, bilateral salpingo-oophorectomy, omentectomy, and lymphadenectomy for suspected ovarian cancer. Microscopic examination revealed a highly cellular tumor composed of small cells with hyperchromatic, round-to-oval nuclei, and scanty cytoplasm. Perivascular pseudorosettes, ependymal rosettes, and extensive necrosis were observed (Figure 3). Diagnosis was confirmed by immunophenotype showing strong positivity to glial fibrillary acidic protein. The tumor tissue also shows strong positivity to estrogen and progesterone receptors.

Patient was treated with low molecular weight heparin (LMWH) 0.6 mL subcutaneous twice a day for five days. Oral anticoagulant warfarin 5 mg per day was started on 3rd day of LMWH with maintenance of the international normalized ratio (INR) within the therapeutic range (2-3) for six months.

There are no standard guidelines for the adjuvant chemotherapy of ovarian ependymomas. Postoperative adjuvant chemotherapy was started with BEP regimen at an interval of 21 days, containing bleomycin 30U IV on days 2, 9, and 16, etoposide 100 mg/m^2^ on days 1–5, and cisplatin 20 mg/m^2^ on days 1–5. In view of tumoral strong positivity to estrogen and progesterone receptors, the patient was put on letrozole 2.5 mg once a day.

After 3 cycles of chemotherapy, CT scan of the abdomen was done which did not show any abnormality. Neck Doppler revealed no thrombus in right internal jugular vein. A total of 6 courses of chemotherapy were completed. Patient is under regular follow up in our clinic with imaging reports for any recurrence of the disease.

## 3. Discussion

Paraneoplastic syndrome is a disease or symptom that is the consequence of the presence of cancer in the body but, unlike mass effect, is not due to the local presence of cancer cells and may precede the diagnosis of malignancy. Treatment is directed towards primary etiology and symptoms with or without immunosuppression. Some of the syndromes may not be reversible. Venous thromboembolism (VTE) and particularly idiopathic VTE may be paraneoplastic phenomena. Thromboembolic events are a major cause of morbidity in cancer patients and may be harbingers of occult malignancy.

Thrombosis of the upper limbs and neck is very rare as compared to lower extremities. Internal jugular vein thrombosis is a very serious event, which can lead to pulmonary embolism and intracranial extension leading to intracranial thrombosis and cerebral edema. Patients usually present with painful swelling in the neck but sometimes may also be asymptomatic [1].

A hypercoagulable or prothrombotic state of malignancy occurs due to the ability of tumor cells to activate the coagulation system. Prothrombotic factors in cancer include the ability of tumor cells to produce and secrete procoagulant (tissue factor, cancer procoagulant, and factor V receptor)/fibrinolytic substances (plasminogen activator and plasminogen activator inhibitor-1, 2) and inflammatory cytokines (IL-1b, TNF-a, and VEGF) and the physical interaction between tumor cell and blood (monocytes, platelets, and neutrophils) or vascular cells. Other mechanisms include nonspecific factors such as the generation of acute phase reactants, abnormal protein metabolism, and hemodynamic compromise (i.e., stasis). In addition, anticancer therapy (i.e., surgery/chemotherapy/hormone therapy) may significantly increase the risk of thromboembolic events by similar mechanisms, for example, procoagulant release, endothelial damage, or stimulation of tissue factor production by host cells [5]. Sometimes thrombosis may be attributed to excessive production of erythropoietin by the kidney secondary to a pressure effect on the ureter by an ovarian tumor.

Cancer alone was associated with a 4.1-fold risk of thrombosis, whereas chemotherapy increased the risk 6.5-fold [6]. The increased risk for venous thrombosis in cancer has been considered an epiphenomenon. Patients who are taking cisplatin based chemotherapy for an advanced ovarian malignancy are associated with venous thrombosis in upto 10% of cases [7]. Tumour cells can directly activate the clotting through two procoagulants: tissue factor (TF) and cancer procoagulant (CP) (Molnar et al., 2007) [8].

Ependymomas are a rare type of glioma. Ependymomas usually arise in the central nervous system, but they can also occur rarely in various extraaxial locations. They develop from the ependymal cells, which line the ventricles (fluid-filled spaces in the brain), and from the central canal of the spinal cord. They can be found in any part of the brain or spine. In children, they are more commonly found in the cerebellum.

Ovarian ependymomas are extremely rare tumors of the ovary with gliomatous differentiation toward ependymal cells that usually arises in the central nervous system. Ependymomas usually develop from neuroectodermal organs. Pure ovarian ependymoma is an extremely rare tumor, and the treatment strategies for this disease have not been established. Differential diagnosis included mainly endometrioid and small cell carcinoma of the ovary. The histologic feature that most facilitated the diagnosis of ovarian ependymoma is the prominence of rosettes and perivascular pseudorosettes. With positive immunohistochemical staining for GFAP, this cellular pattern is quite characteristic of an ependymoma [9].

Although rare, primary ovarian ependymoma must be kept in mind in the differential diagnosis of ovarian tumors. As such there are no standard guidelines for the management of ovarian ependymomas. Administration of etoposide-based chemotherapy along with cytoreductive surgery is a potential standard treatment for advanced ovarian ependymoma [10]. A combination of cisplatin and etoposide (EP) or bleomycin, etoposide, and cisplatin (BEP) is preferred because of a lower relapse rate and shorter treatment time. BEP regimen includes bleomycin (30 units/week); etoposide (100 mg/m^2^/day for days 1–5); and cisplatin (20 mg/m^2^/day for days 1–5) for 4–6 cycles. Patients who do not respond to BEP may benefit from the following as salvage therapy (TIP). TIP includes cisplatin 35 mg/m^2^ days 1, 2, and 3, ifosfamide 2 gm/m^2^ days 2, 3, and 4, and taxol 135 mg/m^2^ day 1 (NCCN guidelines). If there is a strong tumoral expression of estrogen and progesterone receptors, an aromatase inhibitor can be initiated [11].

Options for the initial treatment of cancer-associated thrombosis include LMWH, unfractionated heparin (UFH), and fondaparinux. Vitamin K antagonists (VKAs) like warfarin have been the mainstay agents for long-term management and secondary prophylaxis of cancer associated thrombosis.

## 4. Conclusion

Ovarian cancer is usually epithelial in origin in most of the geriatric patients. Thus the diagnosis of ovarian ependymoma is an unusual occurrence. Further IJVT was the first symptom that developed in this patient. After ruling out other common conditions for thrombosis in this age group, this seems to be a paraneoplastic presentation of ovarian malignancy that preceded the diagnosis of ependymoma by 2 months. An extensive review of the literature did not reveal many cases of ovarian ependymoma. To the best of our knowledge this is the first case report in the world literature with an association between internal jugular vein thrombosis and bilateral ovarian ependymoma.

## 5. Learning Points

1. Knowledge of tumor thrombosis and paraneoplastic hypercoagulability is very important.
2. Histopathologic diagnosis and treatment of ovarian ependymoma are challenging.
3. Surgery and etoposide-based chemotherapy are the mainstay of treatment.
